# FAK/PYK2 inhibitors defactinib and VS-4718 enhance immune checkpoint inhibitor efficacy

**DOI:** 10.1186/2051-1426-3-S2-P354

**Published:** 2015-11-04

**Authors:** Jennifer Ring, Yajuan Li, Irina Shapiro, Yan Wang, David Weaver, Jonathan Pachter

**Affiliations:** 1 Verastem Inc., Needham, MA, USA

## 

Although durable responses to single agent immune checkpoint inhibitors have been reported, additional approaches are needed to extend this therapeutic benefit to a greater proportion of cancer patients. Accordingly, substantial efforts are ongoing to identify agents that can augment T cell-mediated killing of tumor cells and potentiate the effects of checkpoint inhibitors. Focal Adhesion Kinase (FAK) and the closely related family member PYK2 are potentially valuable targets in this regard due to the roles of these protein kinases in regulating key cellular populations in the tumor microenvironment. In addition to the potency of the small molecule FAK/PYK2 inhibitors defactinib (VS-6063) and VS-4718 to target cancer stem cells, we have also reported that these agents inhibited monocyte-derived macrophages, decreased IL-6 production from macrophages *in vitro*, and reduced tumor-associated macrophages in xenograft models.

We now report that defactinib and VS-4718 stimulate proliferation of CD8+ cytotoxic T cells. This is in distinct contrast to other protein kinase inhibitors, such as the SRC inhibitor dasatinib and the MEK inhibitor trametinib, which potently impair the proliferation of CD8+ cytotoxic T cells. Using primary human CD8+ T cells isolated from healthy donors, both FAK/PYK2 inhibitors dose-dependently increased proliferation of CD8+ T cells in the presence of anti-CD3/anti-CD28 coated beads.

Based on the observed enhancement of CD8+ T cells and previously noted inhibition of tumor-associated macrophages, we investigated whether FAK/PYK2 inhibitors potentiate the anti-tumor efficacy of an anti-PD-1 monoclonal antibody in syngeneic mouse tumor models. Mice bearing established MC38 colorectal tumors were treated with VS-4718 in combination with an anti-PD-1 antibody. Combination of VS-4718 with anti-PD-1 extended the median overall survival to 42 days relative to 21, 25 and 28 day median overall survival with vehicle control, single agent anti-PD-1 and single agent VS-4718, respectively. Moreover, on day 56, 30% of mice treated with the VS-4718/anti-PD-1 combination were alive in contrast to the vehicle control, single agent VS-4718, and single agent anti-PD-1 groups in which no mice survived. Studies are currently underway to better understand the immune cell changes in these tumors following VS-4718 and anti-PD-1 combination therapy.

These data provide a rationale for clinical trials in cancer patients to test whether a FAK/PYK2 inhibitor in combination with an immune checkpoint inhibitor could increase the breadth of responsive tumor types, increase the number of responders, and confer a more durable anti-tumor response.

